# Image quality and radiation dose comparison for abdominopelvic CT studies performed using photon-counting CT and dual-energy CT: a clinical study

**DOI:** 10.1093/bjro/tzag012

**Published:** 2026-05-22

**Authors:** Areej Aljabal, Lakshmi Ananthakrishnan, Neha Yadu, Anish Goel, Eric Zeikus, Richard Ahn, Liqiang Ren, Yin Xi, Xinhui Duan

**Affiliations:** Department of Radiology, UT Southwestern Medical Center, Dallas, TX 75390, United States; Department of Medical Imaging, Ministry of National Guard - Health Affairs, Riyadh, Saudi Arabia; King Abdullah International Medical Research Center, Riyadh, Saudi Arabia; Department of Radiology, UT Southwestern Medical Center, Dallas, TX 75390, United States; Department of Radiology, UT Southwestern Medical Center, Dallas, TX 75390, United States; UT Southwestern Medical Center, Dallas, TX 75390, United States; Department of Radiology, UT Southwestern Medical Center, Dallas, TX 75390, United States; Department of Radiology, UT Southwestern Medical Center, Dallas, TX 75390, United States; Department of Radiology, UT Southwestern Medical Center, Dallas, TX 75390, United States; Department of Radiology, UT Southwestern Medical Center, Dallas, TX 75390, United States; Department of Radiology, UT Southwestern Medical Center, Dallas, TX 75390, United States

**Keywords:** photon-counting CT, image quality, radiation dose, abdominopelvic exams

## Abstract

**Objectives:**

To compare radiation dose and image quality of abdominopelvic CT studies performed on dual-energy energy-integrating detector CT (EID-CT) and photo-counting CT (PCCT) for the same patient cohort with clinically aligned protocols.

**Methods:**

An IRB-approved retrospective cohort study included 49 patients who underwent portal venous phase abdominopelvic CT exams using both dual-energy CT and PCCT scanners within a maximum interval of 3 months. Images reconstructed included virtual monoenergetic images at 50, 60, and 70 keV and mixed images. CTDI_vol_ values were recorded to assess radiation dose. Regions of interest were drawn in the 6 anatomical locations for quantitative analysis. The overall image quality, contrast enhancement, image noise, and presence of artifacts were evaluated using a 5-point Likert scale.

**Results:**

No significant differences were observed in image quality scores between PCCT and EID-CT for most comparisons. Calculated contrast-to-noise ratios (CNR) were significantly higher on PCCT except for 70 keV. PCCT demonstrated a significantly lower radiation dose, with an average reduction of 20% ± 15%, up to 51%.

**Conclusion:**

For abdominopelvic CT exams with our institutional protocol settings, PCCT has demonstrated a significant reduction in radiation dose while maintaining similar image quality as EID-CT.

**Advances in knowledge:**

With clinically aligned protocols for the same cohort of patients, the results provided robust evidence for understanding the benefits of PCCT. It also provided a possible pathway to transit from EID-CT to PCCT in the abdominopelvic imaging.

## Introduction

Despite advances in CT, the core detector design of an energy-integrating detector (EID) has remained relatively unchanged until recent innovations. A new CT technology, photon-counting CT (PCCT), has emerged that utilizes a novel detector system based on a photon-counting detector (PCD). PCCT fundamentally changes how X-ray photons are detected and processed. Unlike conventional EIDs, which accumulate the total deposited energy from X-ray photons over a specific time interval, PCDs can resolve individual X-ray photon events using high-speed detectors and electronics.^1^^,^^2^

The PCCT detection mechanism relies on direct conversion of each individual photon into separate electronic pulses and then they are assigned into adjustable energy bins for spectral and non-spectral imaging. Energy-integrating detector-CT detectors are integrating the total deposited energy from all impinging photons, causing photons with all energy levels to be summed together indiscriminately.^1^ This inability to discriminate photon energies limits EID-CT’s capability for material differentiation and contrast enhancement.^3^ Photon-counting CT offers multi-energy or energy-resolving spectral capabilities natively with every scan, unlike many current EID-CT systems, which require the pre-selection of a dual-energy mode before acquiring images to achieve spectral capabilities.^4^ The distinctive detection physics of PCCT improves spatial resolution, eliminates the electronic noise in the projection data, and thus provides greater potential for image quality improvement compared to EID-CT.^5^

Both EID-based dual-energy CT (DECT) and PCCT scanners can generate virtual monochromatic images (VMIs),^6^ which represents a significant advancement in clinical abdominal imaging. Several comparative studies have evaluated both radiation dose and image quality in abdominal CT exams between EID-CT and PCCT. Most have shown that PCCT spectral capabilities improve the signal-to-noise ratio (SNR) or contrast-to-noise ratio (CNR), which is crucial for tasks such as abdominal imaging where soft tissue contrast is limited.^7–9^

However, studies comparing PCCT-based VMI directly with EID-CT-based VMI in abdominal imaging are limited. Existing investigations involved either comparing only polychromatic images generated from both scanners,^10^ or comparing PCCT VMIs at different keV levels with only linear-blended images from EID-CT.^11–14^ More recent comparative studies directly compared the VMIs of PCCT vs EID-CT; however, both studies we found were performed on phantoms using abdominopelvic contrast-enhanced CT protocols.^15^^,^^16^

This study aims to directly compare the radiation dose and image quality of VMIs reconstructed from PCCT with those from EID-CT in abdominopelvic contrast-enhanced CT examinations for the same patient cohort.

## Methods

### Study population

This retrospective study, approved by the Institutional Review Board, identified adult patients (≥18 years) who underwent contrast-enhanced portal venous phase abdominopelvic CT on both PCCT (NAEOTOM Alpha, Siemens Healthineers) and dual-source DECT (SOMATOM Force, Siemens Healthineers) between October 2023 and March 2024 and the 2 scans for the same patient were within a 3-month interval. Patient demographic characteristics are summarized in the “Results” section.

### Image acquisition and reconstruction

All abdominopelvic CTs were performed in the portal venous phase after administering a weight-based dose of iohexol (Omnipaque 350, GE Healthcare) at 0.45 g iodine/kg body weight with the injection rate of 3 mL/s. The details of image acquisition parameters and reconstruction settings are in Table 1.

**Table 1:** Scan protocol and reconstruction parameters for PCCT and EID-CT scanners.

		PCCT	EID-CT
Acquisition parameters	Scan mode	QuantumPlus	Dual energy
	Tube potential (kV) [kV range]	CARE kV [120, 140]	100/Sn150 kV
	AEC* (CARE Dose4D)	IQ 147	Quality Ref. mAs 147/75
	Spiral pitch factor	0.8	0.6
	Rotation time (s)	0.5	0.5
	Slice thickness (mm)	5	5
	Detector configuration (mm)	144 × 0.4	192 × 0.6
	Reference CTDIvol (mGy)	8.3	8.6
Reconstruction parameters	Reconstruction algorithm with strength level	QIR-3	ADMIRE-3
	Convolution kernels	Qr44	Qr44
	Image type	VMI 50, 60, and 70 keV	Mixed Image, VMI 50, 60, and 70 keV

Acquisition parameters were selected to achieve comparable radiation dose and image quality for a reference-sized patient rather than direct equivalence of vendor-specific settings. For EID-CT, QRM values of 147 and 75 were applied to the 100 kV and Sn150 kV tubes, while PCCT used an IQ level of 147. Although these parameters are not directly equivalent, they produced similar scanner-reported CTDIvol values based on the AEC-specific body reference size (8.6 mGy for EID-CT and 8.3 mGy for PCCT). Patient-size-dependent dose behavior was evaluated separately using water equivalent diameter (WED).

Automatic exposure control (CARE Dose4D) was applied using system-specific configurations: PCCT used automatic kV selection with average modulation strength, while EID-CT used QRM-based modulation with a strong profile. These settings reflect routine clinical protocols in our institution. Accordingly, parameters were not directly matched but selected to achieve clinically comparable image quality. The system-specific AEC configurations reflect each scanner’s clinical protocol as deployed in routine practice at our institution. The modulation strength settings for each scanner were adopted through iterative clinical experience, informed by institutional preference, patient population characteristics, and radiologist feedback. These settings were therefore preserved in this study to enable evaluation of scanner performance under genuine real-world conditions.

A total of 7 image datasets were reconstructed for each patient: 50, 60, and 70 keV VMIs for both EID-CT and PCCT, and linear-blended (mixed) EID-CT images using Syngo.Via (Siemens Healthineers, Version VB60A). In both EID-CT and PCCT, VMIs of 50, 60, and 70 keV were reconstructed to facilitate a paired comparison between EID-CT and PCCT systems, for the following reasons. The VMI of 70 keV is commonly used as a replacement for 120 kV images due to its similar CT numbers and reduced artifacts.^6^ VMIs at energy levels of 50 and 60 keV have been shown to have improved CNRs and diagnostic confidence in contrast-enhanced exams.^14^^,^^17^ In the current clinical PCCT, VMIs are the standard images used for interpretation, and the vendor recommends a VMI of 60 keV for contrast-enhanced soft tissue evaluation. A linear-blended dataset (mixed images) was also generated from the EID-CT since it is the default dataset typically used for clinical interpretation as it simulates a 120 kV CT image (and closely resembles a 70 keV VMI reconstruction).

To facilitate a comprehensive evaluation, 2 types of comparisons were performed. First, a direct head-to-head comparison was conducted between matched virtual monoenergetic images (VMIs) reconstructed at identical energy levels (50, 60, and 70 keV) from both PCCT and EID-CT systems. This comparison was designed to provide a technically controlled assessment under equivalent reconstruction conditions.

Second, an additional comparison was performed between PCCT VMI at 70 keV and EID-CT linear-blended (mixed) images. This comparison was included to reflect routine clinical practice, as mixed images represent the default dataset used for interpretation in our institution and in many clinical settings, approximating conventional 120 kV imaging. These 2 comparison approaches were considered complementary, enabling evaluation of both technical equivalence and clinical applicability. T3D images are not routinely used in our clinical practice and are less commonly used in clinical practice at other institutions as well, which is supported by the recent protocol consensus statement from the Society of Abdominal Radiology.^18^

### Image quality assessment

For each patient, a total of 7 datasets (3 different VMIs per scan, plus linear-blended EID-CT) were reconstructed from the 2 scanners, and qualitative and quantitative image analyses were performed. For qualitative analysis, 3 board-certified radiologists, blinded to the scanner and image type, independently reviewed each reconstruction. Radiologists performed a blinded review of the images, which were presented in a randomized sequence in the clinical PACS system (Sectra IDS7, Sectra AB). Radiologists were allowed to freely scroll through each dataset and adjust window/center settings. Radiologists assessed each reconstruction on a 5-point Likert scale for overall image quality, contrast enhancement, image noise, and artifacts. Table 2 shows the Likert scale scoring criteria.

**Table 2:** Criteria for the 5-point Likert scale evaluating overall subjective image quality.

Score	Artifacts	Contrast enhancement	Noise	Overall score
1	Extensive artifacts/non-diagnostic	None—diagnostic exam	Diagnosis impossible	Unacceptable/non-diagnostic
2	Severe artifacts/low confidence in diagnosis	Low confidence in diagnosis	Major noise/low confidence in diagnosis	Poor/low confidence in diagnosis
3	Minor artifacts/diagnosis possible	Acceptable for diagnosis	Moderate/sufficient for diagnosis	Acceptable/adequate/fair
4	Minimal artifacts	Good	Mild/diagnosis not influenced	Good
5	None/no significant artifacts	Excellent	None	Excellent/optimal

**Figure 1:**
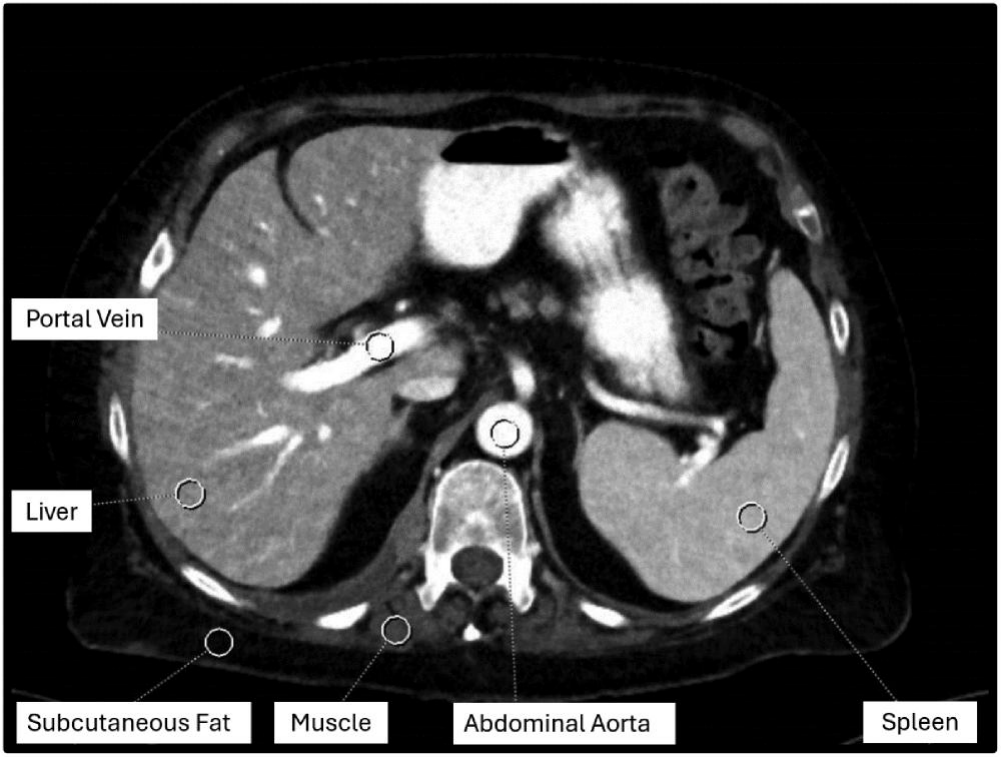
Locations of the 6 regions of interest evaluated in our study (including liver, spleen, portal vein, abdominopelvic aorta, subcutaneous fat, and muscle), as displayed on an axial contrast-enhanced abdominopelvic PCCT at 50 keV.

For quantitative analysis, 6 regions of interest (ROIs) on axial CT images were drawn within the liver, spleen, portal vein, abdominopelvic aorta, subcutaneous fat, and muscle (Figure 1) by an abdominopelvic radiology fellow. The mean CT number and image noise (quantified as the SD, $\sigma_{\mathrm{ROI}}$) for each ROI was recorded. Contrast-to-noise ratios were subsequently computed using the equation below:


$${\mathrm{CNR}}_{\mathrm{ROI}}=\frac{{\mathrm{HU}}_{\mathrm{ROI}}-{\mathrm{HU}}_{\mathrm{fat}}}{\sigma_{\mathrm{fat}}}$$

${\mathrm{HU}}_{\mathrm{ROI}}$ , CT number of each region of interest (ROI or organ); ${\mathrm{HU}}_{\mathrm{fat}}$, CT number of fat (as background); $\sigma_{\mathrm{fat}}$, SD of CT number measurement in fat.

### Radiation dose assessment

Our institution utilizes a dedicated software platform (Radimetrics, Bayer HealthCare) for radiation dose tracking and analysis. Radiation dose metrics, including CTDI_vol_ and patient-specific WED values, were retrieved from the Radimetrics platform for each scan.

### Data analysis

The primary endpoints were the mean differences of overall quality score between PCCT and EID-CT for each setting and mean differences of CNR between PCCT and DECT for each setting and stratified by anatomical location. Secondary endpoints included mean differences for other qualitative scores and noise. Contrast-to-noise ratio was selected as a primary quantitative endpoint because it more directly reflects contrast detectability in contrast-enhanced CT, whereas SNR does not account for differences between tissues.

For image quality, Likert scale scores, CNR, and image noise were analyzed using a linear mixed model (LMM) to account for the variability introduced by multiple readers and image quality parameters. To compare image quality between PCCT and EID-CT at each VMI setting plus PCCT 70 keV vs mixed images from EID-CT, differences in mean values of image quality parameters were estimated, along with their SEs. Sidak p value adjustment was implemented to control family-wise error rates in multiple pairwise comparisons. Inter-reader agreement was calculated using Gwet’s AC1 statistics. Paired t-tests were used to compare qualitative values. To assess the potential confounding effect of patient size on image quality comparisons, the interaction between scanner type and WED was evaluated for noise and CNR using the same linear mixed model framework. Data analysis was performed using R software (Version 4.4.3; R Core Team) with the packages readxl, tidyverse, gtsummary, gt, irrCAC, lme4, and emmeans, package. All statistical tests were 2-tailed. The significance level was set at *P* < .05.

## Results

### Patient demographics

**Figure 2:**
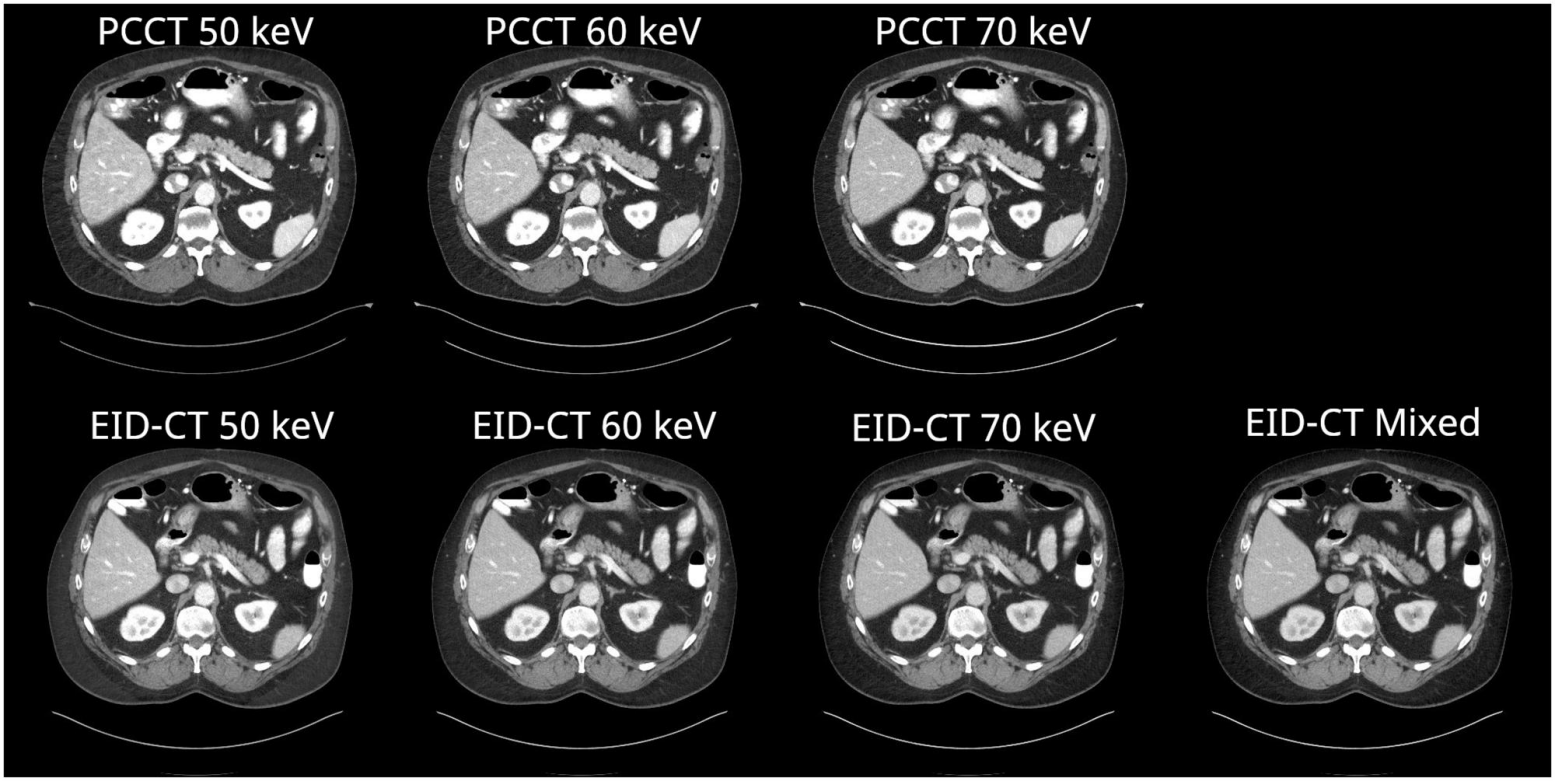
An example case (age 64, male) with all the image types included in the study. The display window and level for each image were individually adjusted to make the image appearing at the same grayscale. The radiation output (CTDIvol) for the EID-CT scan was 21.8 and 12.8 mGy for PCCT (41% dose reduction).

A total of 49 patients met the inclusion criteria. The cohort included 59% males (*n* = 29) and 41% females (*n* = 20). The age ranged from 27 to 87 years, with a mean age of 60 ± 15 years. The mean body weight was 79 ± 19 kg (range: 49-132 kg), and the mean body mass index (BMI) was 26.6 ± 5.5 kg/m^2^ (range: 16.8-47.0 kg/m^2^). One example case is shown in Figure 2.

No significant differences in patient size metrics were observed between the 2 scans within the same patients, as expected for this intra-patient study design.

### Qualitative image analysis

The small mean differences and corresponding SDs (0.04 ± 0.05) indicate minimal discrepancies between the scanners in terms of artifacts, contrast enhancement, image noise, as well as the overall score. The detailed results are shown in Table 3.

**Table 3:** Comparison of qualitative analysis for artifacts, contrast enhancement, image noise, and overall image quality between 2 scanners.

Comparison of quality scores between scanners by VMI (energy level)				
	PCCT	EID-CT	Difference (SE)	Sidak adjusted *P-*value
Artifacts				
50 keV	4.56 ± 0.64	4.52 ± 0.64	0.05 (0.05)	.8
60 keV	4.75 ± 0.52	4.74 ± 0.55	0.01 (0.05)	>.9
70 keV	4.8 ± 0.49	4.80 ± 0.52	0.01 (0.05)	>.9
PCCT (70 keV) vs EID-CT (mixed)	4.80 ± 0.49	4.73 ± 0.57	0.07 (0.05)	.3
Contrast enhancement				
50 keV	4.99 ± 0.12	4.91 ± 0.42	0.07 (0.04)	.3
60 keV	4.96 ± 0.20	4.86 ± 0.34	0.10 (0.04)	.11
70 keV	4.76 ± 0.43	4.43 ± 0.54	0.33 (0.04)	<.001
PCCT (70 keV) vs EID-CT (mixed)	4.76 ± 0.43	4.39 ± 0.53	0.37 (0.04)	<.001
Image noise				
50 keV	4.08 ± 0.67	4.35 ± 0.59	−0.27 (0.05)	<.001
60 keV	4.59 ± 0.52	4.68 ± 0.48	−0.09 (0.05)	.3
70 keV	4.78 ± 0.42	4.80 ± 0.44	−0.02 (0.05)	>.9
PCCT (70 keV) vs EID-CT (mixed)	4.78 ± 0.42	4.81 ± 0.41	−0.03 (0.05)	>.9
Overall image score				
50 keV	4.29 ± 0.63	4.41 ± 0.57	−0.12 (0.05)	.061
60 keV	4.64 ± 0.55	4.65 ± 0.51	−0.01 (0.05)	>.9
70 keV	4.72 ± 0.48	4.63 ± 0.50	0.09 (0.05)	.3
PCCT (70 keV) vs EID-CT (mixed)	4.72 ± 0.48	4.61 ± 0.50	0.12 (0.05)	.086

The analysis revealed “Good” reader agreements for the overall image score (AC1 = 0.52, 95% CI, 0.47-0.57), and noise perception (AC1 = 0.54, 95% CI, 0.49-0.58), “Very Good” agreement for the contrast enhancement (AC1 = 0.75; 95% CI, 0.71-0.78) and artifacts (AC1 = 0.71; 95% CI, 0.67-0.75).

### Quantitative image analysis

**Figure 3:**
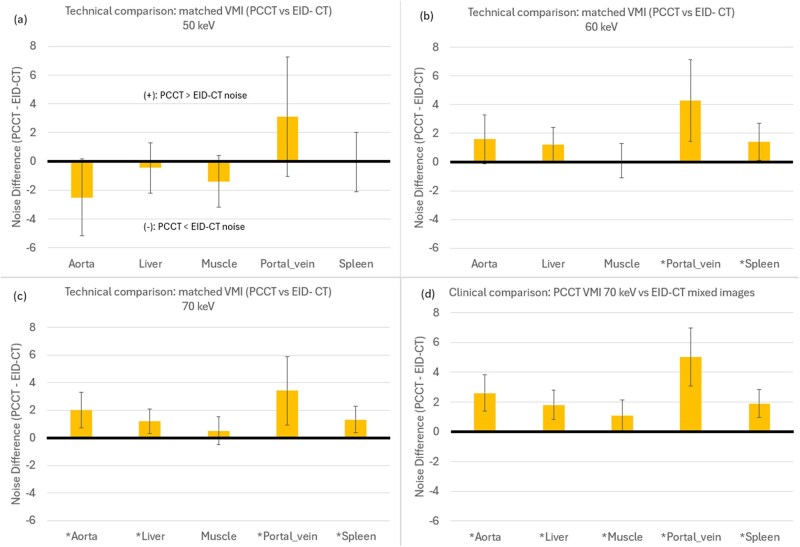
Noise comparison of virtual monoenergetic images (VMIs) at 50 (a), 60 (b), and 70 keV (c) acquired using EID-CT and PCCT. Matched VMI comparisons (PCCT vs EID-CT at identical keV levels) represent the primary technical evaluation under equivalent reconstruction conditions. In addition, PCCT 70 keV (d) VMI was compared with EID-CT mixed images to reflect routine clinical interpretation workflows, where mixed images approximate conventional 120 kV imaging. Statistically significant differences between the groups are indicated by asterisks (*P < .05). Error bars represent 95% CIs.

**Figure 4:**
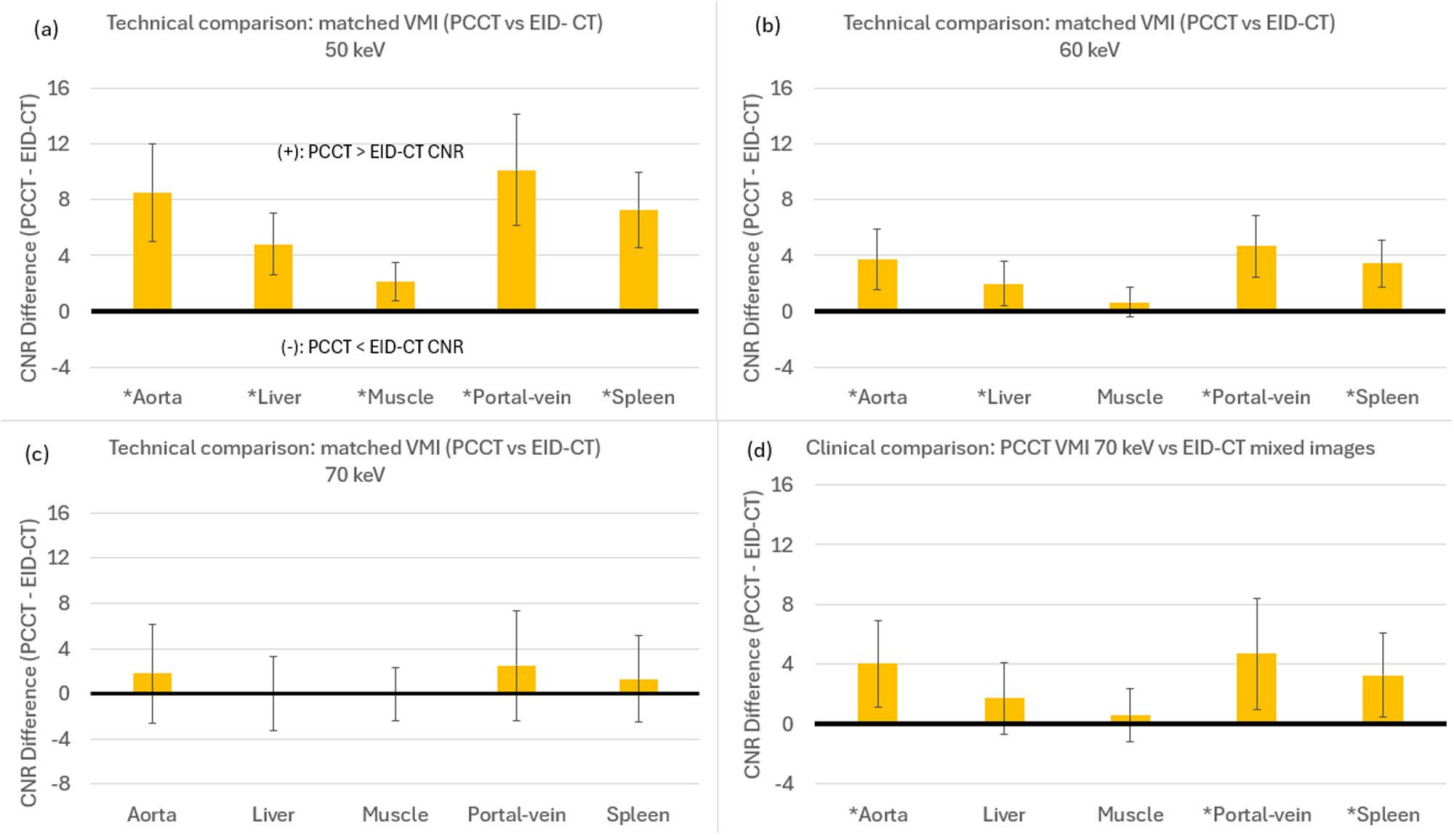
Contrast-to-noise ratio (CNR) comparison of virtual monoenergetic images (VMIs) at 50 (a), 60 (b), and 70 keV (c) acquired using EID-CT and PCCT. Matched VMI comparisons (PCCT vs EID-CT at identical keV levels) provide a controlled technical assessment of image quality. Additionally, PCCT 70 keV (d) VMI was compared with EID-CT mixed images to reflect clinical practice, where mixed images are commonly used for routine interpretation. Statistically significant differences between the groups are indicated by asterisks (*P < .05). Error bars represent 95% CIs.

The noise and CNR comparison at all VMI levels is illustrated in Figures 3 and 4.

Small mean differences and SE in noise data (−0.3 ± 1.3 HU) suggested negligible differences at 50 keV. For 60 keV, noise at portal vein and spleen became significantly higher for PCCT. PCCT 70 keV images demonstrated significantly higher image noise compared to both EID-CT 70 keV and mixed images in most structures, as shown in Figure 3.

The CNR at 50 and 60 keV was significantly higher for PCCT compared to EID-CT for all anatomical structures, with the exception of muscle at 60 keV. Contrast-to-noise ratio at 70 keV PCCT was higher than EID-CT mixed images in some but not all anatomic locations, as shown in Figure 4.

Detailed noise and CNR are provided in the Supplementary Material.

### Radiation dose assessment

**Figure 5:**
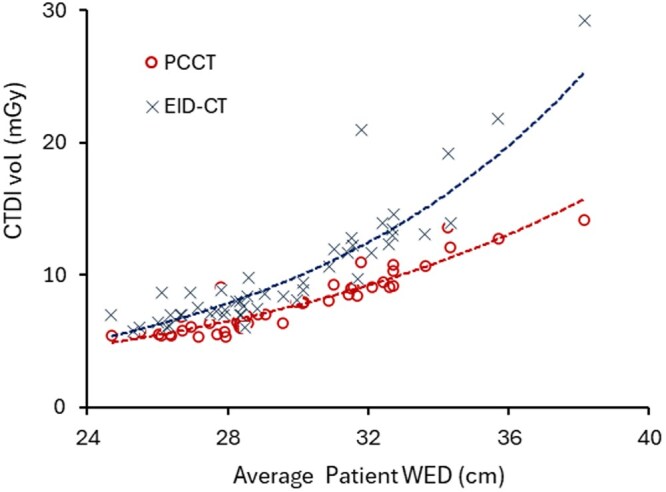
Radiation dose in PCCT and EID-CT as a function of patient size (WED). The relationship between radiation dose (CTDIvol) and patient size, quantified by water equivalent diameter (WED, cm), is shown for both PCCT and EID-CT. At lower WED values, both scanners exhibit comparable radiation doses. However, as WED increases, EID-CT demonstrates a more rapid increase in radiation dose compared to PCCT.

Photo-counting CT demonstrated a lower radiation dose compared to EID-CT, with an average reduction of 20%. The per-patient dose difference (EID-CT − PCCT) ranged from −0.45 to 15.0 mGy, corresponding to a dose reduction range of −7% to 51%. The mean radiation dose for PCCT scans was 7.8 ± 2.3 and 10.2 ± 4.7 mGy for the EID-CT (*P* < .05). Moreover, the results show a relationship between radiation dose reduction and average patient size, quantified by WED. At small average patient sizes, both scanners exhibit similar dose values. Both scanners exhibit a trend where the radiation dose increases as the average patient size increases (Figure 5), as expected. However, with larger patient size, PCCT dose increased to a lesser degree compared to EID-CT, with PCCT exhibiting up to a 51% dose reduction compared to EID-CT for patients of larger body habitus. To further characterize this, the interaction between scanner type and WED was evaluated for noise and CNR. The difference in noise slope between PCCT and EID-CT was statistically significant at 50 keV (slope difference = 0.944, SE = 0.341, *P* = .008) and 60 keV (slope difference = 0.895, SE = 0.243, *P* < .001), confirming that image noise in PCCT increases more steeply with patient size. In contrast, the difference in CNR slope between scanners was non-significant across all anatomical structures and all keV levels (all *P* ≥ .2), indicating that the CNR comparison remained consistent across the full range of patient sizes and was not meaningfully confounded by the differing AEC settings.

## Discussion

This study provides a comprehensive evaluation and compares the radiation dose and image quality of abdominopelvic CT studies performed on both dual-energy EID-CT and PCCT systems within the same patient cohort. Our study found that, using our institutional protocol settings, PCCT offers comparable or improved image quality with lower radiation doses compared to EID-CT. PCCT exhibited higher dose reduction compared to EID-CT for larger patient sizes.

A key strength of our methodology lies in its paired comparative analysis, where PCCT and EID-CT performance was evaluated by assessing image noise, CNR, and quality scores on VMIs reconstructed at identical, pre-defined energy levels (50, 60, and 70 keV) across both systems within the same patient cohort. The comparison also evaluated overall radiation dose with an emphasis on the impact of patient size on dose reduction capabilities. This approach enabled a direct comparison of the 2 CT scanners, which have different radiation detection mechanisms, thereby reducing inter-patient variability and allowing for a more accurate assessment of their respective strengths and limitations.

Two complementary comparison approaches were employed to evaluate PCCT and EID-CT performance from both technical and clinical perspectives. The comparison between matched VMIs at identical energy levels provides a controlled assessment of image quality under equivalent reconstruction conditions, allowing for a more direct evaluation of system performance. In contrast, the comparison between PCCT VMIs and EID-CT mixed images was intended to reflect real-world clinical workflows, where mixed images are typically used for routine interpretation.

These comparisons address different but complementary questions—technical equivalence vs clinical applicability—and should be interpreted accordingly. The inclusion of both approaches allows for a more comprehensive understanding of how PCCT performs relative to EID-CT, both under standardized conditions and in routine clinical use. Importantly, conclusions regarding scanner performance were primarily based on matched VMI comparisons, while cross-type comparisons were used to provide additional clinical context.

While CTDIvol reflects scanner radiation output, it is not directly comparable across patients because automatic exposure control adjusts dose according to patient size. To provide a more precise assessment, this study utilized WED as a standardized size metric, and evaluated CTDIvol for the portal venous phase, conducting a per-patient dose analysis. Our study found that PCCT achieved an average dose reduction of 20% ± 15%, compared to the doses used for the EID-CT system.

Wrazidlo et al.^10^ investigated radiation dose differences between PCCT and EID technologies within an oncologic patient cohort undergoing abdominal imaging, a similar patient population as our study. Their research, based on comparing only polychromatic images (T3D images), concluded that PCCT offers the potential for substantial dose reduction while preserving image quality similar to that of EID-CT. Building upon their comparison, our study significantly expands the assessment beyond just polychromatic images by incorporating an analysis of performance across multiple virtual monoenergetic imaging levels, offering a more detailed evaluation of the PCCT capabilities.

The results in our study illustrate the feasibility of dose reduction in PCCT imaging and is concordant with prior studies.^7^^,^^19^^,^^20^ Furthermore, our findings align with those from other studies in various body regions^20–22^ emphasizing PCCT’s dose-efficacies capabilities throughout the body.^23^^,^^24^ Emerging research has shown that PCCT systems can significantly reduce radiation dose, ranging from 56% to 85%, compared to EID-CT systems in sinus and temporal bone imaging, all while maintaining image quality.^25^

Acquisition protocols were not identical, particularly in AEC settings. PCCT used moderate modulation, whereas EID-CT used stronger modulation, reflecting routine clinical practice and system-specific optimization.

Therefore, the observed dose reduction reflects combined effects of detector technology and protocol design rather than hardware alone. Because AEC depends on patient size, these differences may also contribute to the greater dose reduction observed in larger patients. While clinically relevant, this limits strict comparability. Prospective studies with harmonized AEC settings are needed to isolate detector effects.

Patients with larger body habitus present unique challenges in CT imaging due to increased radiation dose requirements and limitations of current CT technologies. While dual-source CT scanners offer the potential for increased total photon flux when operated in single-energy mode, a major limitation is the reduced field-of-view in one of the X-ray tubes,^6^ resulting in incomplete anatomical coverage by both tubes, which is particularly problematic in abdominopelvic imaging. In addition, the user must choose between operating the scanner in single-energy mode at higher tube voltage (optimal for patients of larger body habitus) vs dual-energy mode, limiting the ability for spectral analysis in patients of larger body habitus.^26^ PCCT solves these limitations by using higher kVs (120 and 140 kV) and a full field-of-view scanning.

In our investigation, PCCT yielded superior CNR compared to EID-CT at 50 keV VMI. This finding is consistent with Higashigaito et al.,^11^ who also performed an intra-individual comparison of abdominal PCCT and EID-CT. They proposed that 50 keV might represent an optimal energy level for PCD-CT VMI. Another intra-individual comparison study was done by Schwartz et al,^27^ which also shows improved CNR from PCCT compared with EID-CT.

This study has several limitations. First, its retrospective, single-center design may limit generalizability. Second, acquisition parameters were not fully matched due to differences in system design, particularly AEC implementation. As AEC depends on patient size, these differences may introduce size-dependent effects and confound attribution of dose reduction to detector technology alone.

Protocols were based on clinical implementation rather than formal optimization using standardized metrics (eg, SNR or CNR), which may limit technical comparability. Future studies with harmonized acquisition parameters are needed to isolate system-specific effects.

## Conclusion

This intra-patient study compares abdominopelvic CT using clinically implemented PCCT and EID-CT protocols. Notably, PCCT achieved comparable image quality with lower radiation dose using standard vendor-recommended settings, while EID-CT relied on institutionally refined protocol settings developed over years of clinical experience. These findings suggest that PCCT’s detector technology may inherently support improved dose efficiency under routine clinical conditions. Further studies with harmonized acquisition parameters are warranted.

## Supplementary Material

tzag012_Supplementary_Data
