# Supplementary material for: Image quality and radiation dose comparison for abdominopelvic CT studies performed using photon-counting CT and dual-energy CT: a clinical study
Source: BJR Open. 2026 May 22;8(1):tzag012. doi: 10.1093/bjro/tzag012 (PMC13259597; doi:10.1093/bjro/tzag012)
Supplement: tzag012_Supplementary_Data [file tzag012_supplementary_data.docx]

Table 1. Noise comparison results

| **Location** | **PCCT** N = 49*^1^* | **EID-CT** N = 49*^1^* | **Difference***^2^* | **95% CI***^2^* | **p-value***^2^* |
| --- | --- | --- | --- | --- | --- |
| **aorta** | | | | | |
| 50 keV | 24.98 ± 7.02 | 27.47 ± 7.09 | -2.5 | -0.17, 5.2 | 0.066 |
| 70 keV | 16.24 ± 4.05 | 14.24 ± 3.07 | 2.0 | -3.3, -0.72 | 0.003 |
| 60 keV | 20.39 ± 5.21 | 18.80 ± 4.01 | 1.6 | -3.3, 0.11 | 0.066 |
| 70 keV vs. Mixed | 16.24 ± 4.05 | 13.63 ± 2.27 | 2.6 | -3.8, -1.4 | <0.001 |
| **Liver** | | | | | |
| 50 keV | 19.51 ± 4.63 | 19.96 ± 4.13 | -0.45 | -1.3, 2.2 | 0.6 |
| 70 keV | 13.63 ± 2.71 | 12.41 ± 1.86 | 1.2 | -2.1, -0.36 | 0.006 |
| 60 keV | 16.24 ± 3.41 | 15.06 ± 2.36 | 1.2 | -2.4, 0.00 | 0.051 |
| 70 keV vs. Mixed | 13.63 ± 2.71 | 11.88 ± 2.06 | 1.8 | -2.7, -0.76 | <0.001 |
| **Muscle** | | | | | |
| 50 keV | 19.47 ± 4.43 | 20.88 ± 5.26 | -1.4 | -0.40, 3.2 | 0.12 |
| 70 keV | 13.92 ± 3.12 | 13.41 ± 2.62 | 0.51 | -1.5, 0.50 | 0.3 |
| 60 keV | 16.31 ± 3.26 | 16.20 ± 3.18 | 0.10 | -1.3, 1.1 | 0.9 |
| 70 keV vs. Mixed | 13.92 ± 3.12 | 12.86 ± 2.40 | 1.1 | -2.1, -0.06 | 0.039 |
| **portal_vein** | | | | | |
| 50 keV | 31.27 ± 15.39 | 28.14 ± 6.71 | 3.1 | -7.3, 1.0 | 0.14 |
| 70 keV | 19.06 ± 7.17 | 15.63 ± 5.94 | 3.4 | -5.9, -0.94 | 0.008 |
| 60 keV | 24.08 ± 10.24 | 19.76 ± 4.91 | 4.3 | -7.2, -1.5 | 0.003 |
| 70 keV vs. Mixed | 19.06 ± 7.17 | 14.10 ± 3.01 | 5.0 | -6.9, -3.0 | <0.001 |
| **Spleen** | | | | | |
| 50 keV | 20.41 ± 6.53 | 20.02 ± 3.79 | -0.04 | -2.0, 2.1 | >0.9 |
| 70 keV | 13.43 ± 3.33 | 11.96 ± 1.70 | 1.3 | -2.3, -0.38 | 0.007 |
| 60 keV | 16.55 ± 4.53 | 14.87 ± 2.40 | 1.4 | -2.7, -0.14 | 0.030 |
| 70 keV vs. Mixed | 13.43 ± 3.33 | 11.41 ± 1.54 | 1.9 | -2.8, -0.96 | <0.001 |
| Abbreviation: CI = Confidence Interval | | | | | |
| *^1^* Mean ± SD | | | | | |
| *^2^* Paired t-test | | | | | |

Table 2 CNR results

| **Location** | **PCCT** N = 49*^1^* | **EID-CT** N = 49*^1^* | **Difference***^2^* | **95% CI***^2^* | **p-value***^2^* |
| --- | --- | --- | --- | --- | --- |
| **CNR-aorta** | | | | | |
| 50 keV | 31 ± 11 | 23 ± 6 | 8.5 | 5.2, 12 | <0.001 |
| 60 keV | 25 ± 6 | 21 ± 6 | 3.7 | 1.6, 5.9 | 0.001 |
| 70 keV | 23 ± 10 | 21 ± 13 | 1.8 | -2.7, 6.2 | 0.4 |
| 70 keV vs. Mixed | 23.2 ± 9.9 | 19.1 ± 5.7 | 4.1 | 1.1, 7.0 | 0.008 |
| **CNR-liver** | | | | | |
| 50 keV | 19.3 ± 6.9 | 14.5 ± 4.2 | 4.8 | 2.6, 7.0 | <0.001 |
| 60 keV | 17.0 ± 4.4 | 15.0 ± 4.5 | 2.0 | 0.45, 3.6 | 0.012 |
| 70 keV | 17.1 ± 7.1 | 17.1 ± 9.0 | 0.00 | -3.3, 3.3 | >0.9 |
| 70 keV vs. Mixed | 17.1 ± 7.1 | 15.3 ± 5.0 | 1.7 | -0.56, 4.1 | 0.13 |
| **CNR-muscle** | | | | | |
| 50 keV | 11.40 ± 4.42 | 9.27 ± 2.81 | 2.1 | 0.74, 3.5 | 0.003 |
| 60 keV | 10.93 ± 3.06 | 10.27 ± 3.16 | 0.66 | -0.42, 1.7 | 0.2 |
| 70 keV | 11.9 ± 5.8 | 11.9 ± 6.5 | -0.03 | -2.4, 2.3 | >0.9 |
| 70 keV vs. Mixed | 11.9 ± 5.8 | 11.2 ± 4.0 | 0.62 | -1.2, 2.4 | 0.5 |
| **CNR-portal_vein** | | | | | |
| 50 keV | 33 ± 13 | 23 ± 7 | 10 | 6.2, 14 | <0.001 |
| 60 keV | 27 ± 6 | 22 ± 6 | 4.7 | 2.5, 6.9 | <0.001 |
| 70 keV | 25 ± 13 | 22 ± 13 | 2.5 | -2.5, 7.4 | 0.3 |
| 70 keV vs. Mixed | 25 ± 13 | 20 ± 6 | 4.7 | 1.1, 8.4 | 0.012 |
| **CNR-spleen** | | | | | |
| 50 keV | 23.4 ± 9.4 | 16.0 ± 4.3 | 7.3 | 4.5, 10 | <0.001 |
| 60 keV | 19.5 ± 5.3 | 16.0 ± 4.5 | 3.4 | 1.7, 5.1 | <0.001 |
| 70 keV | 18.9 ± 9.1 | 17.5 ± 9.5 | 1.3 | -2.5, 5.1 | 0.5 |
| 70 keV vs. Mixed | 18.9 ± 9.1 | 15.6 ± 4.3 | 3.2 | 0.50, 6.0 | 0.022 |
